# Blood Vessel Extraction in Color Retinal Fundus Images with Enhancement Filtering and Unsupervised Classification

**DOI:** 10.1155/2017/4897258

**Published:** 2017-08-03

**Authors:** Zafer Yavuz, Cemal Köse

**Affiliations:** Karadeniz Technical University, Trabzon, Turkey

## Abstract

Retinal blood vessels have a significant role in the diagnosis and treatment of various retinal diseases such as diabetic retinopathy, glaucoma, arteriosclerosis, and hypertension. For this reason, retinal vasculature extraction is important in order to help specialists for the diagnosis and treatment of systematic diseases. In this paper, a novel approach is developed to extract retinal blood vessel network. Our method comprises four stages: (1) preprocessing stage in order to prepare dataset for segmentation; (2) an enhancement procedure including Gabor, Frangi, and Gauss filters obtained separately before a top-hat transform; (3) a hard and soft clustering stage which includes K-means and Fuzzy C-means (FCM) in order to get binary vessel map; and (4) a postprocessing step which removes falsely segmented isolated regions. The method is tested on color retinal images obtained from STARE and DRIVE databases which are available online. As a result, Gabor filter followed by K-means clustering method achieves 95.94% and 95.71% of accuracy for STARE and DRIVE databases, respectively, which are acceptable for diagnosis systems.

## 1. Introduction

**Figure 1:**
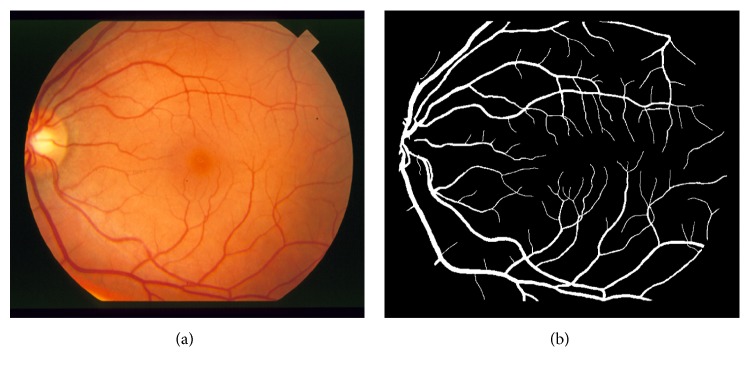
An example of color retinal fundus image (a) and manually segmented binary vessel map (b).

Computer-aided diagnosis (CADx) systems use visioning and analysis techniques such as retinal fundus imaging, ultrasonography (USG), and computer tomography (CT) in recent years. Retinal fundus images with high resolution could help ophthalmologists to diagnose diseases automatically by extracting blood vessels, the optic disc, and macula [1]. Fundus images provide us many features such as retinal vessels, the optic disc, and macula. An example of a retinal fundus image can be seen in Figure 1(a). Retinal vessels which are spread out to the whole retina have a significant role in the diagnosis of various retinal diseases such as diabetic retinopathy, glaucoma, arteriosclerosis, and hypertension. Additionally, retinal blood vessels are main determiners to locate other major retinal structures (optic disc and macula) and they provide many measurable features for the diagnosis of ophthalmologic diseases. For this reason, retinal vasculature extraction is important for the diagnosis and treatment of retinal diseases. Retinal blood vessel extraction is a process to get binary vessel map where retinal blood vessels are labelled as logic 1 (white) and retinal background pixels are labelled as logic 0 (black) or vice versa. An example of binary vessel map labelled by an expert manually is shown in Figure 1(b). The ideal purpose of this study is getting binary vessel image by an automatic computerized algorithm.

Retinal vasculature extraction has some challenges such as pathological diseases and noises observed in the retinal images. Also, it is observed that retinal images have low contrast between blood vessels and the retinal background. Various methods are proposed to accomplish these challenges in the literature. The vessel extraction solutions could be divided into two categories as rule-based and machine learning methods. In rule-based methods, a pixel is labelled as vessel according to some predefined criteria. These methods include two-dimensional matched filter response (2D MFR) [1, 2], morphology-based approaches [3, 4], and vessel-tracking methods [5, 6]. The machine learning methods include supervised and unsupervised classification. In supervised methods, a pixel is labelled as vessel or background by using some features which differentiate vessel from retinal background. These methods are usually called as specially designed classifiers and include k-nearest neighbors (k-NN) [7], support vector machines (SVM) [8], Bayesian decision rule [9], and artificial neural networks [10]. In unsupervised methods, a clustering schema such as hard (K-means) [11] and soft (Fuzzy C-means) [12] clustering is used to decide if a pixel belongs to a vessel or retinal background.

One of the first studies about retinal vasculature extraction is proposed by Chaudhuri et al. [1]. They configured a matched filter using Gauss function, because a vessel profile matches with the inverted Gaussian function. In their method, the Gaussian kernel is obtained firstly and, then, they apply the rotated versions of the Gaussian kernel to the green channel of a color retinal fundus image by convolving with twelve rotated versions of 2D Gaussian (15° of angular resolution) and the maximum value with respect to the angle is selected as maximum filter response. Then, Otsu's thresholding algorithm is used and the final vessel map is obtained. Another study using a two-dimensional matched filter response (2D MFR) is suggested by Hoover [2]. After the vessel enhancement procedure with 2D MFR, they apply a threshold probing method to determine local threshold and get binary vessel map. The method was tested on manually labelled vessel images which is referred as STARE databases [13]. A local thresholding method for retinal vasculature extraction is proposed by Jiang and Mojon [14]. They divide the gray level intensities into sublevels. Candidate vessel images are obtained by selecting pixels between maximum and minimum values of each sublevel. Then, a verification procedure is applied and the verified sublevels are selected. Finally, the binary vessel map is acquired by combining these verified sublevels before a postprocessing stage. They tested their method on STARE database and gave the results [14]. Gabor-based vessel segmentation study is suggested by Soares et al. [9] in 2006. In their study, they used some features extracted from multiscale Gabor wavelet transform and then Bayesian classification with these features is performed. Afterwards, the pixels in the retinal image are classified as vessels or nonvessels. STARE [13] and DRIVE [15] databases are used for testing, and obtained results are presented. A texture-based vessel segmentation method which used a bank of Gabor energy filters is proposed by Bhuiyan et al. [16]. Fuzzy C-means clustering method based on features obtained from texture properties is used to classify retinal image pixel vessel or nonvessel. DRIVE database is used in this study, and 84.37% sensitivity is achieved in their study. Another study about vessel segmentation is proposed by Kande et al. [17]. Gaussian-based matched filtering is used for vessel enhancement. To classify the retinal pixels as vessel or background, an unsupervised Fuzzy C-means clustering method is utilized to obtain final vascular tree. They achieve an area under the receiver operating characteristic (ROC) curve of 95.18% and 96.02% on DRIVE and STARE databases, respectively. Lupaşcu and Tegolo suggested another retinal vessel segmentation schema using unsupervised clustering [18]. They use some pixels' features extracted from the output of different filters and transform them to self-organizing maps. K-means clustering methods are used to obtain the final vessel network. An accuracy of 94.59% success is achieved on DRIVE database. Dey et al. [19] proposed a method of blood vessel segmentation using Fuzzy C-means clustering. In their study, a gray level conversion from RGB images is performed before adaptive histogram equalization. Then, a background subtraction procedure is done for feature extraction and Fuzzy C-means clustering is utilized to get binary vessel map. Another study proposed by Nguyen et al. used a basic and multiscale line detector with various lengths and scales for enhancement. They achieve 93.24% and 94.07% accuracy value for STARE and DRIVE datasets, respectively [20]. Budai et al. enhanced the original Frangi filter [21] to achieve higher accuracy and fewer processing time and obtain about 94% average success on STARE and DRIVE images in the same year [22]. A method based on fuzzy logic is proposed by Sharma and Wasson [23]. In this method, the retinal image is filtered with a high-passed filter and then a few different fuzzy rules are applied to the output of the high-passed image to extract blood vessels. The method is tested on DRIVE database. A cake filter is employed by Bao et al. [24] to segment retinal blood vessels which uses the real component fusion in order to separate vessel pixels from the retinal background pixels. Then, a self-adaption thresholding is used to obtain final vessel map. In another study, Mapayi et al. presented a local adaptive thresholding method based on gray level cooccurrence matrix and reported the average accuracy values of 95.11% and 95.10% on DRIVE and STARE databases, respectively [25]. A three-stage algorithm includes high-pass filtering, major vessel segmentation, and fine vessel segmentation with Gaussian mixture model (GMM) classifier proposed by Roychowdhury et al. They have achieved segmentation accuracy of 95.2% and 95.15% on DRIVE and STARE, respectively [26]. Some of the latest studies about vessel segmentation are made by Oliveira et al. [12], Rani et al. [27], and Shah et al. [28]. Oliveira et al. used filter combinations that use weighted mean and median ranking of enhancement filters which are matched filter, Gabor wavelet filter, and Frangi filter. Then, an unsupervised classification method was employed in order to segment final vessel map [12]. Rani et al. presented a vessel segmentation algorithm using two-dimensional matched filters, supervised classification techniques (support vector machines and tree bagger), and reported 95.01% and 93.71 of accuracy on STARE and DRIVE databases, respectively [27]. Shah et al. extracted 24 features from a color retinal fundus image and then trained a linear minimum squared error (LMSE) classifier to separate vessels from the background. They achieved 94.79% of accuracy on DRIVE database [28].

Although there are many approaches for retinal blood vessel segmentation, there is still room to get further improvement and to enhance the performance on different images, especially on pathological images. In this study, we propose a novel blood vessel extraction approach based on image enhancement techniques and unsupervised clustering methods include K-means and Fuzzy C-means methods. This paper is organized as follows. The image databases are introduced in Section 2.1.  Section 2.2 gives the preprocessing operations, and Section 2.3 gives the vessel enhancement procedures which contain the feature extraction steps. Clustering phase is described in Section 2.4. The postprocessing step is given in Section 2.5. Results are presented and discussion is done in Section 3. Conclusion is in Section 4.

## 2. Materials and Methods

**Figure 2:**
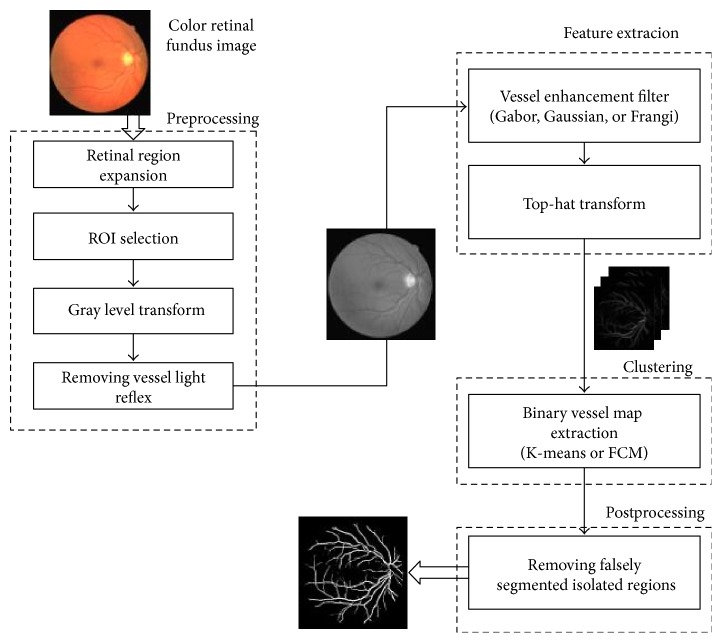
The block diagram of the method.

In this paper, a novel automatic blood vessel segmentation method is performed in color retinal fundus images. A vessel enhancement operation including several matched filters such as Gabor, Gauss, and Frangi is utilized after a preprocessing step. After that, top-hat transform is applied for further enhancement of blood vessels. Then, binary vessel map is obtained by using hard and soft clustering methods (Fuzzy C-means and K-means). Finally, falsely isolated pixels are removed as a postprocessing step. The block diagram of the method is given in Figure 2().

### 2.1. Materials

Various publicly image databases are available online for retinal fundus image analysis utilized by many authors. In this paper, we use two commonly used image databases which are called as structured analysis of the retina (STARE) [13] and digital retinal image for vessel extraction (DRIVE) [15] to test our method.

The STARE database consists of 20 fundus images (ten of them have various pathological cases). The images have been captured with a TopCon TRV-50 digital fundus camera at 35° field of view (FOV). The dimensions of the images are 700 × 605 pixels with 24 bits (8 bits per each color channel). All images in this database have corresponding manually segmented versions in which the pixels are labelled as vessel or nonvessel by two observers. The second observer labelled thin vessels much more. We use the images labelled by the first observer as ground truth data for performance evaluation. There are no region of interest (ROI) masks in this database.

Secondly, the DRIVE database contains 40 images (seven of them have various pathological cases). The images have been captured with a Canon CR5 nonmydriatic 3CCD digital retinal fundus camera at 45° field of view (FOV). The dimensions of the images are 768 × 584 pixels with 24 bits (8 bits per each color channel). All images in this database also have corresponding manually segmented versions. The images have been divided into two parts as training and testing. Each part contains 20 images. We use testing images for performance evaluation. There are corresponding region of interest (ROI) masks in this database.

### 2.2. Preprocessing

#### 2.2.1. Selection and Expansion of Region of Interest (ROI)

Retinal region is circular even though fundus images are rectangular. Therefore, retinal region masks must be utilized in order to select the region of interest in fundus images. There are corresponding masks in DRIVE database whereas many databases such as STARE do not contain any mask to select retinal region. Thus, retinal region mask might be segmented automatically for a general framework.

**Figure 3:**
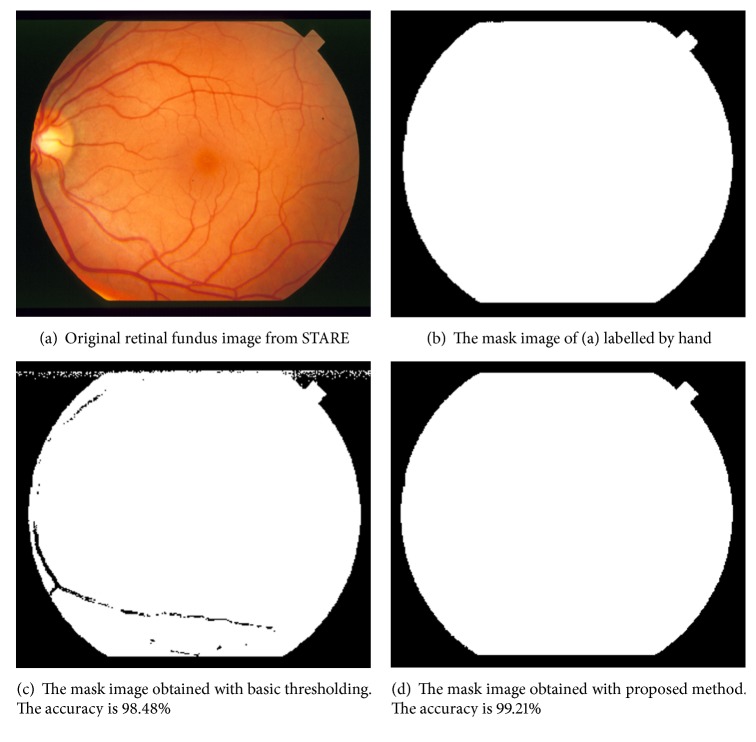
An example of retinal region selection operation.

Many studies apply a basic threshold value to the intensity image in order to select retinal region, but some pixels belonging to vessels or dark regions in the retina could be segmented as out of the retina. In this study, we use not only a basic threshold but also additional criteria which are difference values between color channels (red, green, and blue) and the average of RGB values. Firstly, we calculate the average of RGB values. Then, the sum of the difference between RGB values is calculated and we decided the mask value according to

(1) $$\begin{array}{c} mask\left(x,y\right)=\left\{\begin{array}{c} 1,\;avg\left(R,G,B\right)<T_{1},\;D<T_{2}\\ 0,\;otherwise, \end{array}\right. \end{array}$$

where *D* = |*R* − *G*| + |*R* − *B*| + |*G* − *B*|. In 1, *T*_1_ and *T*_2_ are the threshold values and determined empirically. For the best performance, *T*_1_ is taken as 40 and *T*_2_ is taken as 50. When we use only a basic threshold, the average accuracy is calculated as 99.04% and the standard deviation is 1.01% and some vessel pixels are classified as mask pixels. An example of this effect is illustrated in Figures 3(c) and 3(d). When we use the criteria mentioned above, the average accuracy is calculated as 99.78% and the standard deviation is 0.25% and none of vessel pixels are classified as mask pixels.

Many vessel enhancement approaches utilize convolution operation with symmetric structured elements. Consequently, unwanted artefacts might occur on the border of the retina. An expansion of ROI is applied in order to solve this problem, after the selection of retinal region. Firstly, the pixel set on the border of the retina is obtained and, then, a pixel reflection operation is performed according to border pixels by taking the symmetric values. Here, the reflection size is set as 6 for the best results.

#### 2.2.2. Gray Level Conversion

There are various gray level conversion methods in order to obtain an intensity image. Some methods might use only one of the color channels such as green channel whereas others use the sum of multiplication such as the color channels with special coefficients. The sum of the color coefficients must be 1 (*c*_r_ *+ c*_g_ *+ c*_b_ = 1) to get intensity as normalized. The general formula is given in

(2) $$\begin{array}{c} I=c_{r}*R+c_{g}*G+c_{b}*B, \end{array}$$

where *I* is the intensity value, *R*, *G*, and *B* are the intensities of red, green, and blue color channels, respectively, and *c*_x_ values are the color coefficients. In many retinal vessel segmentation methods, the green channel of the color image is taken as gray level intensity because the maximum contrast is obtained with this channel (*c*_r_ = 0, *c*_g_ = 1, and *c*_b_ = 0). Some other methods could use the average of RGB values (*c*_r_ = 1/3, *c*_g_ = 1/3, and *c*_b_ = 1/3) or special gray level transform coefficients such as *c*_r_ = 0.3, *c*_g_ = 0.59, and *c*_b_ = 0.11. In this paper, we selected the color channel coefficients as *c*_r_ = 0.1, *c*_g_ = 0.7, and *c*_b_ = 0.2 by using a training set from image databases (STARE and DRIVE). Some details could be found in our previously done study [29].

#### 2.2.3. Vessel Light Reflex Removal

**Figure 4:**
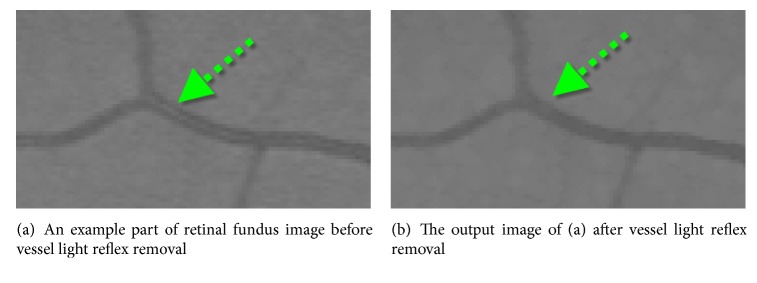
An example to vessel light reflex removal.

Since retinal blood vessels have lower brightness compared to the retina, their intensities are darker than other retinal pixels but a brighter strip could be observed on the center of some retinal blood vessels. To remove this effect, a gray level morphological opening operation is applied to the intensity image with three-pixel diameter disc [10]. This operation is illustrated in Figure 4.

### 2.3. Feature Extraction

In this stage, feature extraction procedure is performed by using several vessel enhancement methods such as Gabor filter response, Gaussian-matched filter response, and Frangi's multiscale vessel enhancement approach in order to enhance retinal blood vessels.

#### 2.3.1. Gabor Filter

**Figure 5:**
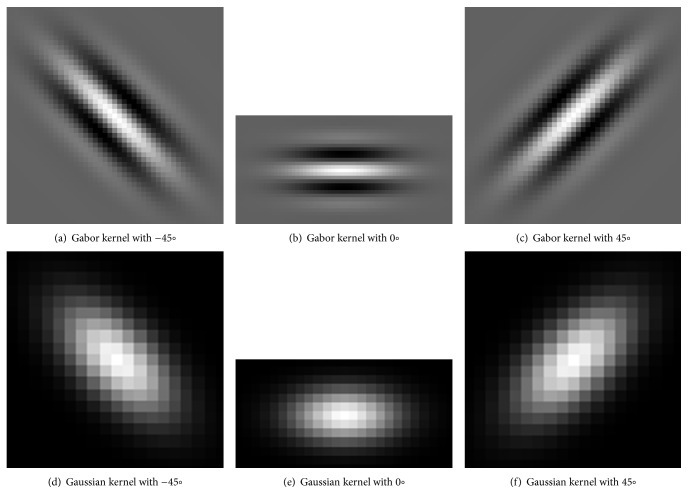
Different orientations of Gabor and Gauss kernels used in vessel enhancement.

Gabor filters are widely utilized in some research areas such as computer vision, pattern recognition, and image analyzing for texture enhancement and feature extraction. A complex Gabor function is constructed by multiplying a Gaussian envelope function with a complex trigonometric function. In many texture enhancement approaches, the real part of complex Gabor function is taken. In this paper, the real part of a two-dimensional Gabor kernel function is used in order to enhance retinal blood vessels. The formula of 2D Gabor kernel function used in this paper is given in

(3) $$\begin{array}{c} g\left(x,y;\lambda ,\theta ,\sigma ,\gamma \right)=\exp\left(-\frac{x^{\prime 2}+\gamma^{2}y^{\prime 2}}{2\sigma^{2}}\right).\cos\left(2\pi .\frac{x^{\prime }}{\lambda }\right), \end{array}$$

where *x*′ = *x*.cos*θ* + *y*.sin*θ*, *y*′ = −*x*.sin*θ* + *y*.cos*θ*, and *g* is the 2D Gabor kernel function. This function has four parameters to control the shape: scale, orientation, wavelength, and aspect ratio. Sigma (*σ*) is the scaling parameter which is the standard deviation of the Gaussian envelope. Theta (*θ*) is the orientation, lambda (*λ*) is the wavelength, and gamma (*γ*) is the aspect ratio. Some examples of Gabor kernels are given in Figure 5() with several orientations and scales. In this study, a Gabor filter bank is obtained by rotating a Gabor kernel with specified parameters to span all possible orientations. Angular resolution is selected as 15°, so 12 different Gabor kernels were obtained. The preprocessed intensity image is convoluted with these 12 Gabor kernels, and the maximum response is selected for each pixel. Consequently, the pixels belong to the retinal blood vessels which are more dominant compared to background pixels. Sample Gabor kernels with different orientations are given in Figures 5(a), and 5(b), and 5(c).

#### 2.3.2. Gaussian-Matched Filter

Two-dimensional Gaussian-based matched filtering was firstly used for retinal blood vessel segmentation by Chaudhuri et al. [1]. Since the retinal cross-sectional vessel profile is similar with a Gaussian shape, a two-dimensional Gaussian-based matching template could be utilized for best approximation to the blood vessels. The mathematical equation of a 2D Gaussian template could be described as

(4) $$\begin{array}{c} G\left(x,y\right)=-\exp\left(\frac{x^{2}+y^{2}}{2\sigma^{2}}\right), \end{array}$$

where *σ* is the standard deviation of the Gaussian function and could be assumed as the scale of the Gaussian filter. *x* and *y* parameters are rotated to span all possible orientations. In this paper, angular resolution has taken as 15° in a similar manner mentioned in Gabor filter. Sample Gaussian kernels are given in Figures 5(d), 5(e), and 5(f) (the aspect ratio of Gaussian is taken as 0.5).

#### 2.3.3. Frangi Filter

**Figure 6:**
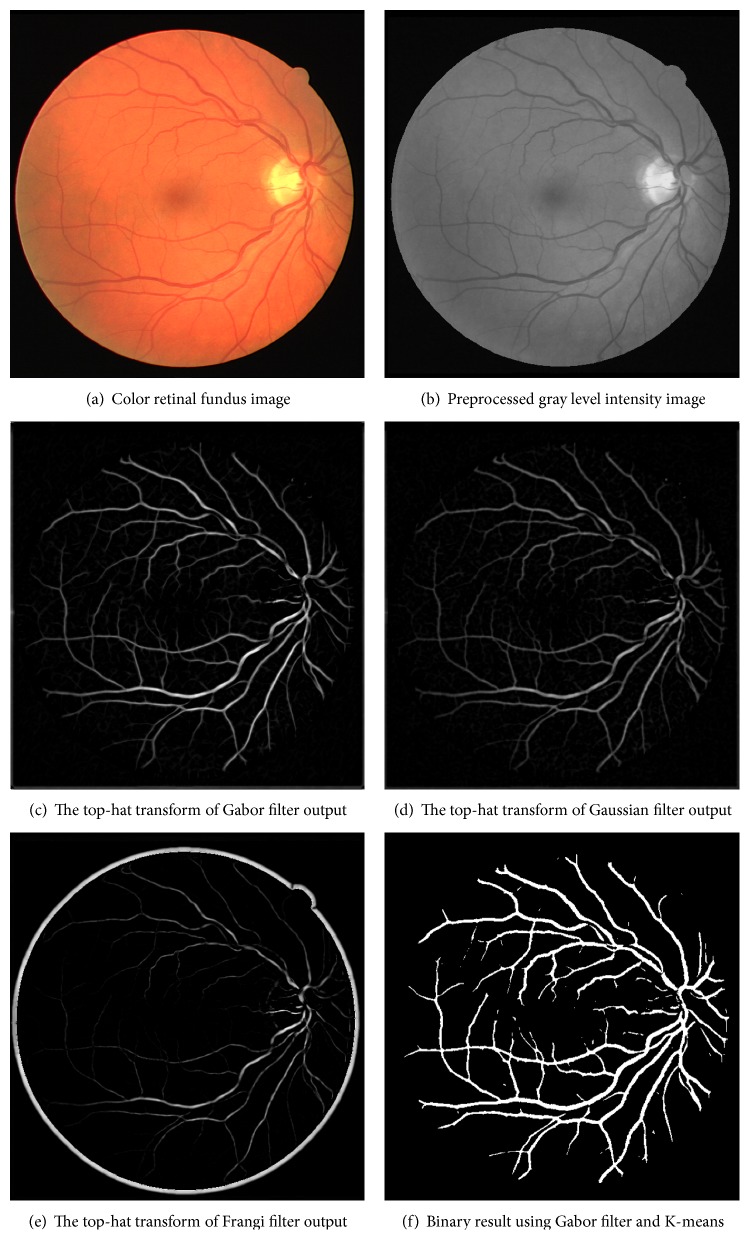
Example results of different filters (Gabor, Gaussian, and Frangi) and binary result.

A multiscale approach for the enhancement of vessel-like structures was suggested by Frangi et al. [21]. They describe a local vesselness likelihood value in their study. According to the method, Hessian matrix of the intensity image which uses the second partial derivatives is obtained and then the eigenvalues of the Hessian matrix are calculated. The Hessian matrix is given in

(5) $$\begin{array}{c} H=\left[\begin{array}{cc} I_{xx} & I_{xy}\\ I_{yx} & I_{yy} \end{array}\right], \end{array}$$

where *I*_*xx*_,  *I*_*xy*_, *I*_*yx*_, and *I*_*yy*_ are the second partial derivatives of the intensity image. The eigenvalues of Hessian matrix *λ*_1_ and *λ*_2_ are calculated and ordered as |*λ*_1_| < |*λ*_2_|. Then, a vesselness likelihood value is obtained according to *V*(*s*) function given in (6) and (7) for each pixel.

(6) $$\begin{array}{c} V\left(s\right)=\left\{\begin{array}{l} 0\;\lambda_{2}>0,\\ \exp\left(-\frac{{R_{B}}^{2}}{2\beta^{2}}\right)\left(1-\exp\left(\frac{S^{2}}{2c^{2}}\right)\right)\;\lambda_{2}\leq 0, \end{array}\right. \end{array}$$

(7) $$\begin{array}{c} R_{B}=\frac{\left|\lambda_{1}\right|}{\left|\lambda_{2}\right|},\;S=\sqrt{{\lambda_{1}}^{2}+{\lambda_{2}}^{2}}, \end{array}$$

where *β* and *c* are the parameters to configure the sensitivity of *R*_B_ and *S* values, respectively. The scale parameter is *s* and takes integer values between 1 and 5. The maximum response of all *V*(*s*) values are selected for each pixel, and the output of the Frangi filter which is shown in Figure 6(e) is obtained.

#### 2.3.4. Top-Hat Transform

Top-hat transform is a morphological operation that is utilized to extract small objects and details by increasing contrast in digital image-processing applications [30]. In this study, the top-hat transform is applied to the output of filters such as Gabor, Gaussian, and Frangi mentioned above. It increases the contrast between blood vessels and retinal background pixels. Top-hat transform is obtained by subtracting the morphologically opened image from the original intensity image. This is illustrated in

(8) $$\begin{array}{c} TH\left(I\right)=I-\left(I\circ nB\right), \end{array}$$

where TH is the top-hat transform image, *I* is the filtered image, *B* is the morphological structure used in gray level opening, and *n* is the scaling parameter of the transform. In the application, we have set the structuring element size as 5 pixels (*n* = 5) with disc shaped for the best performance.  Figure 6 shows the top-hat transform of different enhancement filters such as Gabor, Gaussian, and Frangi.

### 2.4. Vessel Segmentation with Clustering

After the feature extraction stage, the binary vessel map must be obtained. A clustering method which classifies the pixels as vessel or nonvessel is implemented for this reason. Clustering methods are divided into two subclasses as hard clustering and soft clustering. We utilized K-means algorithm as hard clustering and Fuzzy C-means (FCM) clustering as soft clustering.

#### 2.4.1. Hard Clustering (K-Means)

K-means method is one of the simplest unsupervised learning algorithms that solve the well-known clustering problem proposed by MacQueen [31] in 1967. We use this unsupervised method to classify enhancement image pixels. *K* value refers the number of regions in the image. In K-means method, each sample (points in this study) is assigned to the nearest centroid (cluster) after an initialization procedure. Then, the centers of each cluster are recalculated and the assignment step is repeated until the system becomes stable (the system is stable if the error is less than a threshold value).

We applied K-means algorithm to segment blood vessels as follows: firstly, retinal images are enhanced with vessel enhancement filters (Gabor, Gaussian, and Frangi) and top-hat transforms. Then, K-means method is applied to the output of the vessel enhancement filters. The parameter *K* (region count) is set to 3. It means that the image is divided into three sets. These sets are the points in which the possibility of vesselness is high, medium, and low, respectively. At the final stage, the regions in which possibility of vesselness is high and medium are combined and the binary vessel map is obtained.

#### 2.4.2. Soft Clustering (Fuzzy C-Means)

In Fuzzy C-means (FCM) clustering algorithm (which is the generalized form of clustering methods), each sample could be a member of more than one set whereas in K-means algorithm, each sample could be a member of only one set. For example, one sample can be a member of one set with 75% proportion and can be a member of another set with 25% proportion in Fuzzy C-means methods. In FCM method, some parameters such as *C* (cluster count), *n* (sample count), and *m* (the exponential weight factor) are initialized and the membership matrix values are determined randomly. Then, the membership matrix is recalculated until the system becomes stable (if the error is less than a threshold value).

Herein, we propose a novel approach to extract blood vessels with Fuzzy C-means clustering with a similar manner mentioned in K-means clustering section. Firstly, retinal images are enhanced with vessel enhancement filters (Gabor, Gaussian, and Frangi) and top-hat transforms. Secondly, Fuzzy C-means method is applied to the output of the vessel enhancement filters. In this study, the parameter *C* (cluster count) is set as 3. We obtain a clustered image with three regions by applying FCM method. In the first region, the possibility of vesselness is high and the possibility of vesselness is lower than the first category in the second region. In the third region, the possibility of vesselness has the lowest values compared to the others. The first and the second regions are assumed as vessels, and the third region is assumed as background.

Many studies in literature select the region count as two for binary classification traditionally. In this study, different region counts such as *c =* 2 and *c =* 4 are also analyzed at this stage in both K-means and Fuzzy C-means clustering. The obtained performance measured with two or four cluster counts are given in Table 1 which proves that three regions give the best performance result.

### 2.5. Postprocessing

Once we get the binary map of retinal blood vessels, some operations must be performed in order to enhance segmentation performance. These operations are called as the postprocessing step. A postprocessing operation which removes the falsely detected isolated pixel regions is applied in this stage. To remove falsely detected isolated vessel regions, all vessel regions in which the area is less than fourteen pixels are reclassified as background pixels. This operation is also called as length filter by some studies in literature. After the postprocessing step, the final binary vessel map is obtained. An example image of final binary vessel map is given in Figure 6(f).

## 3. Experimental Results and Discussion

In this study, we applied our method on two publicly available online databases known as STARE and DRIVE. As we mentioned in Section 2.1, STARE database has 20 fundus images and DRIVE database has 40 fundus images that were divided into two subsets: the training image set (20 images) and the testing set (the remaining 20 images). We tested our method on all 60 images and utilized the second observer's labelling as ground truth data for comparison. Firstly, some secondary metrics such as true positive (TP), true negative (TN), false positive (FP), and false negative (FN) are calculated as follows:

- True positive (TP): the count of vessel pixels correctly classified as vessel
- True negative (TN): the count of background pixels correctly classified as background
- False positive (FP): the count of background pixels incorrectly classified as vessel
- False negative (FN): the count of vessel pixels incorrectly classified as background.

When we get these measures by using the binary vessel map which is the output of our method and the ground truth data which is manually segmented by an observer, we calculate the sensitivity (true positive rate), specificity, and accuracy measures to measure performance according to (9), (10), and (11). These metrics are commonly used for the evaluation of vessel segmentation methods in literature.

(9) $$\begin{array}{c} Sensitivity=\frac{TP}{TP+FN}\times 100, \end{array}$$

(10) $$\begin{array}{c} Specificity=\frac{TN}{TN+FP}\times 100, \end{array}$$

(11) $$\begin{array}{c} Accuracy=\frac{TP+TN}{TP+TN+FP+FN}\times 100. \end{array}$$

Parameter setting of filters used in feature extraction phase is as follows: Gaussian filter (two-dimensional matched filter) has only one adjustable parameter which is the standard deviation (*σ*) of the function. We choose standard deviation as *σ* = 1 for images in DRIVE database and *σ* = 3 for images in STARE database. Gabor filter has three adjustable parameters which are standard deviation (*σ*) of Gaussian envelope, wavelength (*λ*) of the trigonometric cosine function, and aspect ratio (*γ*) of the Gabor kernel. We choose standard deviation as *σ* = 2, wavelength as *λ* = 10, and aspect ratio as *γ* = 0.5 for the best performance results. On the other hand, Frangi filter has three adjustable parameters which are standard deviation (*σ*) and the tuning parameters *β* and *c*. We choose standard deviation *σ* = 0.5, 1.0, 1.5, …, 5.0, *β* = 4, and *c* = 15. In clustering phase, K-means and Fuzzy C-means clustering methods have one adjustable parameter which is the cluster size. We evaluated the methods with various cluster sizes which are 2, 3, and 4.

The average results obtained by using the images from STARE and DRIVE databases are given in Tables 1–3. In Tables, TPR refers to true positive rate, SPE refers to specificity, and ACC refers to accuracy.  Table 2 shows the average results calculated with the pixels in the whole images by using various filters (two-dimensional matched filter, Gabor filter, and Frangi filter) and clustering methods (K-means and Fuzzy C-means) mentioned above. Gabor filter followed by FCM clustering gives better performance with the images from DRIVE database, and Gabor filter followed by K-means clustering gives better performance with the images from STARE database. For a general approach, Gabor followed by K-means is selected as it gives the best results in average.

The obtained experimental results are compared to the other methods in Table 3, and binary vessel image is illustrated in Figure 6(f). It could be observed that the illustrated binary vessel network given in Figure 6(f) is good and has satisfactory result for visual inspection. The last column in Table 3 shows that the performance metrics are calculated whether in the ROI or in the whole image. It means that the mask image for the retina is considered or not. NA indicates that any information is not available. The proposed method performs 95.94% of accuracy on STARE and 95.71% of accuracy on DRIVE databases where the whole image pixels are considered. The accuracy values obtained by using the mask image (in ROI) are also given for a fair comparison. The results measured in ROI are 94.37% and 94.00% on STARE and DRIVE databases, respectively. Additionally, it is given the specificity (SPE) value in Table 3 that the higher specificity means less noisy results. The proposed approach in this paper outperforms better performance than the most of the other studies with the specificity (the specificity on DRIVE database exceeds all of the other studies). The obtained specificity results are 98.16% and 99.05% on STARE and DRIVE databases, respectively.

Table 1 shows the results obtained by using various cluster sizes such as two, three, and four. As we select the ROI pixels automatically, we consider whole image pixels in the retinal image for performance evaluation. The cluster size is selected as three, because the best performance values are obtained with this value. As seen in Table 1, 95.94% of accuracy for STARE database and 95.71% of accuracy for DRIVE database are obtained using Gabor filter and K-means clustering with cluster size three (*c* = 3).

The obtained experimental results show us some conclusions. First of all, the obtained results are comparable with the literature and give less noisy vessel image (higher specificity) as shown in Table 3 and Figure 6(f). Several vessel enhancement filters are applied, and Gabor filters give better results since they are more successful to highlight local features. In other words, Gabor filters bring out less artefacts compared to others. The results also showed that the parameters tuned for the best performance are different on different image databases. Fuzzy C-means method is soft clustering approach which is a generalization form of hard clustering (K-means clustering). It is expected that soft clustering approaches will give better results than hard clustering. However, this assumption is not true in all situations. The fuzzification of the systems does not give better performance results in every time. In literature, clustering approaches use two clusters in order to classify each pixel as blood vessel pixels or background pixels. This study shows that classification with three clusters is more successful than two or four clusters. Increasing the cluster numbers causes oversegmentation, and decreasing the cluster number causes undersegmentations. This situation is shown in Table 1.

To calculate computational complexity, we have considered core operations which are vessel enhancement filters (Gauss, Gabor, and Frangi) and clustering (K-means and FCM), because every algorithm has some pre- or postprocessing. First of all, some necessary parameters should be defined. Let *n* be the pixel counts in one retinal image. The big *O*(.) notations of submethods are as follows:

- Gauss filter: *O*(*n*.*m*^2^.*a*), where *m* is the height or width of kernel Gauss filter and *a* is the angular resolution parameter (if *a* is 12, then the angular resolution will be 180/12 = 15).
- Gabor filters: *O*(*n*.*m*^2^.*a*), similar to Gauss filter.
- Frangi filter: this filter has three main subprocesses.
  - The Hessian matrix calculation: *O*(*n*.*w*^2^), where *w* is the maximum expected vessel width.
  - Eigenvalue analysis: *O*(*n*), one process for each pixel.
  - Maximum value calculation: *O*(*n*.*N*), where *N* is the number of images.
- K-means clustering: *O*(*n*.*c*.*x*), where *c* is the cluster size and *x* is the iteration number.
- Fuzzy C-means clustering: *O*(*n*.*c*^2^.*t*), where *c* is the cluster size and *t* is the iteration count. It is calculated as a membership matrix despite one-dimensional distance vector in K-means. Calculating membership value needs extra *c* iteration unlike K-means method.

As Gabor filter followed by K-means clustering methods are selected for the best performance, it can be assumed that the big *O* notation of proposed method is *O*(*n*.*m*^2^.*a*) + *O*(*n*.*c*.*x*).

The method which is developed in this paper is implemented using the Matlab® tool. The developed software is evaluated on a Microsoft Windows® 10 personal computer which has 3.3 GHz CPU and 4 GB random access memory. The processing time for a one-color retinal fundus image takes about 9 seconds in average which is acceptable for real-time applications. A comparison is given in Table 4. Some methods use supervised classification and need training operation which requires more time. The running times of these studies which used classification methods could be increase if the training operation is included.

## 4. Conclusion

Retinal blood vessel segmentation approaches could be divided into two categories as rule-based and machine learning methods. We have presented a novel approach based on several blood vessel enhancement and unsupervised classification (clustering) methods within the latter. A preprocessing operation is performed to obtain retinal mask instead of using manually labelled one followed by vessel enhancement filtering. Then, we used two clustering schemas as hard clustering (K-means) and soft clustering (Fuzzy C-means) for pixel classification. In order to test the proposed system, two publicly available retinal fundus image databases (STARE and DRIVE) are used and experimental results are given as sensitivity, specificity, and accuracy.

One of the main contribution of the proposed segmentation method is taking cluster size *c* as three instead of two which we have seen mostly in literature. As given in tables above, higher accuracy values are obtained by increasing cluster size *c*. In traditional clustering methods, the cluster size had been taken as two. However, this paper reveals that taking cluster size as three could provide better performance in any case. The second important feature of our method is using automatically segmented masks for retinal region (region of interest) instead of manually labelled ones. This provides us to produce an automatic solution for a general purpose without any need to manually label retinal mask. The next significant feature of developed system is using unsupervised classification approach which provides us to segment retinal blood vessels without any training operation. Additionally, Gabor filter followed by K-means clustering is a new combination of methods and relatively better than the others. Hard clustering (K-means) schema gave us better segmentation results than soft clustering (FCM). This means that the fuzzification of the systems does not give better performance results in every time. In the future work, we aim to study the measurements of segmented retinal vessels and integrate them with our vessel segmentation method.

## Figures and Tables

**Table 1:** The effect of different cluster numbers to the performance results using hard and soft clustering after Gabor filter.

	TPR	SPE	ACC
*STARE images*			
Gabor + FCM with 2 clusters	51.59	99.34	95.74
Gabor + FCM with 3 clusters	75.38	96.83	95.18
Gabor + FCM with 4 clusters	87.06	89.79	89.53
Gabor + K-means with 2 clusters	50.37	99.39	95.70
Gabor + K-means with 3 clusters	**68.69**	**98.16**	**95.94**
Gabor + K-means with 4 clusters	84.30	92.05	91.44
*DRIVE images*			
Gabor + FCM with 2 clusters	54.60	99.01	93.34
Gabor + FCM with 3 clusters	67.76	98.59	95.87
Gabor + FCM with 4 clusters	54.50	99.02	93.34
Gabor + K-means with 2 clusters	54.51	99.01	93.33
Gabor + K-means with 3 clusters	**61.02**	**99.05**	**95.71**
Gabor + K-means with 4 clusters	54.44	99.02	93.33

**Table 2:** The obtained average results from STARE and DRIVE databases in whole image (cluster size is 3).

	TPR	SPE	ACC
*STARE images*			
Gabor filter + FCM	75.38	96.83	95.18
Gauss filter + FCM	59.20	97.07	94.16
Frangi filter + FCM	57.62	98.79	95.71
Gabor filter + K-means	68.69	98.16	**95.94**
Gauss filter + K-means	70.24	97.09	95.16
Frangi filter + K-means	58.98	98.63	95.67
*DRIVE images*			
Gabor filter + FCM	67.76	98.59	**95.87**
Gauss filter + FCM	74.29	97.02	95.00
Frangi filter + FCM	69.27	98.39	95.82
Gabor filter + K-means	61.02	99.05	**95.71**
Gauss filter + K-means	61.79	98.75	95.50
Frangi filter + K-means	68.83	98.43	95.83

**Table 3:** The obtained average results from STARE and DRIVE databases in ROI compared with some other methods (NA indicates “not available”).

	TPR	SPE	ACC	In ROI/whole image
*STARE images*				
Proposed method (Gabor + K-means)	**70.86**	**97.07**	**94.37**	In ROI
Proposed method (Gabor + K-means)	68.69	98.16	**95.94**	In whole image
Hoover [2]	67.51	95.67	92.67	In whole image
Jiang and Mojon [14]	—	—	93.37	NA
Staal et al. [7]	—	—	95.16	In ROI
Nguyen et al. [20]	—	—	93.24	NA
Budai et al. [22]	58.00	98.20	93.86	NA
Oliveira et al. [12] (FCM)	80.49	95.92	94.46	NA
Bao et al. [24]	78.12	96.12	96.24	NA
Mapayi et al. [25]	76.26	96.57	95.10	NA
Roychowdhury et al. [26]	77.20	97.30	95.10	NA
Rani et al. [27]	74.19	97.47	95.01	NA
*DRIVE images*				
Proposed method (Gabor + K-means)	**67.79**	**97.86**	**94.00**	In ROI
Proposed method (Gabor + K-means)	61.02	99.05	**95.71**	In whole image
Chaudhuri et al. [1]	—	—	87.73	In whole image
Xu and Luo [32]	77.60	—	93.28	In ROI
Staal et al. [7]	—	—	94.42	In ROI
Nguyen et al. [20]	—	—	94.07	NA
Budai et al. [22]	64.40	98.70	95.72	NA
Oliveira et al. [12] (FCM)	91.06	94.31	94.02	NA
Mapayi et al. [25]	73.13	97.24	95.11	NA
Roychowdhury et al. [26]	72.50	98.30	95.20	NA
Rani et al. [27]	72.60	96.86	93.70	NA
Shah et al. [28]	72.05	98.14	94.79	NA

**Table 4:** A comparison of running times with existing studies on STARE and DRIVE databases.

	Average running times		System
	STARE	DRIVE	
Proposed method	9.1 s	8.6 s	3.3 GHz CPU, 4 GB RAM
Hoover [2]^∗^	5 min	—	Sun SPARC station
Jiang and Mojon [14]^∗^	8–36 s	8–36 s	600 MHz PC
Staal et al. [7]	15 min	15 min	1 GHz CPU, 1 GB RAM
Nguyen et al. [20]	2.5 s	2.5 s	2.4 GHz CPU, 2 GB RAM
Budai et al. [22]	1.31 s	1.04 s	2.3 GHz CPU, 4 GB RAM
Roychowdhury et al. [26]	6.7 s	3.11 s	2.6 GHz CPU, 2 GB RAM
Rani et al. [27]	3.26 s	2.58 s	3.4 GHz CPU, 4 GB RAM

^∗^The results are reported from cited articles.
